# Report of a Case of a Long Standing Ulcer, from Which Were Extracted Three Teeth, Resembling Those of the Human Jaw

**Published:** 1833

**Authors:** James C. Finley


### Art. III.—*Report of a Case of a long standing Ulcer, from which were extracted three Teeth, resembling those of the Human Jaw.* By James C. Finley, M. D.

In April, 1832, I was requested to visit Harrison ——, about 19 years of age, who had labored since the third year of his age, with a troublesome ulcer upon the abdomen. Upon examination, I found an ulcer about two inches below the original situation of the umbilicus. The external opening was small, but upon examination with the probe, it was found to extend downwards about two inches, under the symphysis pubis, and to occupy about the same extent from side to side. The sinus appeared to be filled by a compressible but very elastic tumor, and there protruded from the orifice a quantity of long black hair, which, from its length, was supposed to grow from the bottom. The bottom of the sinus, when examined with the probe, was endued with great sensibility; and when irritated, produced a desire to evacuate the bladder and a painful sensation in the glans penis. There was constantly discharged a thin and very offensive fluid that rendered the situation of the patient very uncomfortable.

The general health of the patient was good, and his appetite and digestion regular, although his complexion was sallow, and his constitution delicate.

An attempt was made to dilate the orifice of the sinus with wax bougies, which were retained in their position by a bandage passing around the body, increased in size as the orifice dilated; but the enlargement went on very slowly on account of the extreme sensibility of the orifice of the sinus and the cartilaginous hardness which its walls assumed. While pursuing this course, a wash of the chloride of lime was daily used; by which the offensive smell of the discharge was removed, and in a few days the hair came out, and never afterwards made its appearance.

When the orifice of this sinus became sufficiently distended to enable us to see to the bottom, it was found to be filled with a sarcomatous tumor, originating from a broad base of cartilage and bone; bleeding profusely when wounded; and, when any part was removed, quickly regenerated again.

At an early period of his childhood, a small sore had formed at the umbilicus, supposed, by his parents to be produced by the bite of a wood-tick, which, instead of healing, had rapidly burrowed into the cellular texture, and, probably, occupied the sheath of the rectus muscle.

About two years before, a physician in Indiana had attempted an operation, the object of which was to lay the sinus open, remove the unnatural growth with which it was filled, and permit it to heal from the bottom. This object was but partially accomplished, in consequence of unanticipated difficulties. The blood flowed very profusely, and the cartilaginous structure which occupied the wall of the sinus next the abdomen, of so dense an organization as to resist the action of the knife, and being more extensive than had been originally supposed, was not entirely removed. The patient being unwilling to submit to a repetition of the operation, an unsuccessful attempt was made to destroy it with caustic, and consequently, the sinus, although diminished in extent one half, remained with all its inconveniences.

Our first project of a cure was to dilate the orifice of the sinus until the whole of its internal surface was exposed to the action of the air, hoping that the looseness of the cellular texture which covers the abdomen would admit of this extension; and, expecting that the secreting surface with which it was lined would then assume the character of the common integuments. In the first of these expectations we were disappointed, for although on the first application of the bougie, dilatation was easily effected; yet, as we proceeded, the cellular texture became exquisitely sensible, much thickened, and acquired a density almost cartilaginous.

Disappointed in this expectation, it was our design to remove the growth which was probably the original cause of this sinus, and which seemed to form the principal obstacle to a cure. This was a task of no little difficulty. The tumor commenced at the orifice with a base extending downwards an inch, and from side to side about two inches, and from this extending so as to fill the whole cavity. The cavity thus filled was irregular in its shape and possessed of great sensibility; and in addition, the tumor when wounded, bled so profusely that if the wound were at all extensive, it could only be checked by filling the whole cavity with lint.

Under these circumstances it was found to be impracticable to remove the whole of the tumor. At different operations, however, the whole of the bony and cartilaginous base upon which it was founded was taken away, and only a small portion of the sarcomatous part left deep in the sinus, and which it was very difficult to remove without subjecting the patient to more pain than he was willing to submit to.

But one other prospect of cure now remained, viz: to cut through the cellular texture which formed the external wall of the sinus and remove so much as would secure the exposure of the whole to the air. This, however, was so much thickened and so sensible that the patient was unwilling to submit to such an operation, and the prospect of a perfect cure was abandoned.

His condition, however, is very much improved. The sinus is dilated very nearly to the bottom, and although it is still necessary to keep up the distention, yet in consequence of its exposure, the discharge is very much diminished, and its offensive nature so far removed, that by daily washing it with the chloride of lime, he is enabled to get along very comfortably.

The examination of the bony structure which was removed presented some very curious phenomena. Two perfectly formed human teeth projected from the surface of the tumor—the first, a dens caninus rather smaller than natural for a tooth of the second growth, but with a root of the natural size—the second a bicus pis perfect in size and formation, and scarcely to be distinguished by the closest scrutiny, from one extracted from the jaw of an adult. These teeth were inserted in a socket formed upon a piece of very firm bone about three-fourths of an inch in length. At a subsequent period two additional pieces of bone were removed, the one about the size and texture of the former; the other nearly round, three-fourths of an inch in diameter, and of a light cellulated texture. These pieces of bone appeared to constitute the basis upon which the tumor was formed, and were so firmly connected with the body as to admit of little motion.

Respecting the origin of this unnatural growth, it is useless to speculate. Whether it was congenital or not, it is impossible to decide. Perhaps the difficulties respecting its formation are diminished by supposing its rudiments to have been formed coeval with conception, and to be analogous in its origin to those rude attempts at organization which we meet in the ovaria of females, and occasionally in other parts of the viscera—and sometimes even in the male.

